# Spectrofluorimetric Estimation of Scopoletin in *Evolvulus alsinoides* Linn. and *Convulvulus pluricaulis* Choisy.

**DOI:** 10.4103/0250-474X.49139

**Published:** 2008

**Authors:** A. Nahata, V. K. Dixit

**Affiliations:** Department of Pharmaceutical Sciences, Dr. Hari Singh Gour Vishwavidyalaya, Sagar-470 003, India

**Keywords:** *Convulvulus pluricaulis*, *Evolvulus alsinoides*, Scopoletin, *Shankhpushpi*, spectrofluorimetric analysis

## Abstract

A simple sensitive spectrofluorimetric method was developed for the analysis of total coumarins calculated as scopoletin in the plants *Evolvulus alsinoides* and *Convulvulus pluricaulis*. The fluorescent nature of scopoletin proved to be of immense value in the development of the spectrofluorimetric method. The excitation and emission wavelengths for scopoletin were 430 nm and 460 nm respectively and the instrument was Shimadzu RF 5301 PC spectrofluorophotometer. The method was validated in terms of linearity, accuracy and precision. The proposed spectrofluorimetric method provides a faster and cost effective qualitative and quantitative control for routine analysis of scopoletin in *Evolvulus alsinoides* and *Convulvulus pluricaulis* and their formulations.

In the traditional system of medicine, the whole herb of *Shankhpushpi* has been in clinical use for centuries. In the Ayurvedic system of medicine, *Shankhpushpi* is considered as *Medhya Rasayana*-meaning a drug, which rejuvenates, maintains, and potentiates intellect and memory^1^. According to Ayurvedic Pharmacopoeia of India, *Shankhpushpi* consists of whole plant of *Convulvulus pluricaulis* Choisy. (CP, family Convulvulaceae). The pharmacopoeial monograph also states in note that in certain parts of India *Clitorea ternatea* Linn. (Family-Papilionaceae) and *Evolvulus alsinoides* Linn. (EA, family- Convulvulaceae) are used as *shankhpushpi*. Whatever may be the source, the drug finds use for its therapeutic effect on brain disorders like insanity, epilepsy, nervous debility and for memory enhancement^1^. Many formulations containing *shankhpushpi* as a single drug or in combination with other drugs are available in Indian market and *shankhpushpi* is vigorously advertised for memory enhancement in print and electronic media in India. To minimize batch variations and to add scientific validity to herbal formulations, it is necessary that like modern drugs, herbal drugs should also be analyzed and proper quality control techniques be developed to verify the quality and quantity of the herbs added in the formulation. Scopoletin is a major chemical constituent of *Convulvulus micophyllus* Sieb ex Spreng (syn: *Convulvulus pluricaulis* Choisy.) and its TLC densitometric estimation has been given in Quality Standards of Indian Medicinal Plants^2^. During the course of our investigations, we found presence of scopoletin in EA. The present report describes a simple, reproducible, sensitive and rapid method of spectrofluorimetric analysis for the qualitative and quantitative estimation of the total coumarins calculated as scopoletin from EA and CP and their formulations.

The most notable physical characteristic of the majority of natural coumarins is the fact that they are fluorescent in UV light^3^–^5^. These compounds are very easily detected, since they give characteristic fluorescent colors in UV light, which are intensified by further treatment with ammonia vapours. Scopoletin is a coumarin, which shows a blue violet fluorescence in UV light^6^.

This fluorescent characteristic of scopoletin was made the basis for its quantitative estimation in various extracts of EA and CP. For this purpose, the technique of spectrofluorimetry was utilized. The utilization of this technique led to the development of a novel method for determining the concentration of total coumarins calculated as scopoletin in *shankhpushpi*.

The spectrofluorimetric study was carried out with a Shimadzu RF 5301 PC spectrofluorimeter, to determine levels of fluorescence in the coumarins in a stationary state. The light source used was a xenon 150 w lamp with an optical system composed of two automatic monochromators, one for excitation and the other for emission of a mesh type to enable a suitably wide selection of excitation and emission wavelength for the coumarins^7^. A quartz cell was used^8^. The detection system comprised of a R 450-01 photomultiplier which transformed the fluorescent radiation emitted by the scopoletin solution in the cell into an electrical signal.

Aerial parts of EA and CP were collected from the village Bhapel in Sagar district, Madhya Pradesh, India in the months of January to March, 2006. The herbs were identified in the Department of Botany, Dr. Hari Singh Gour Vishwavidyalaya, Sagar and preserved with voucher specimen numbers HBD/12030 and HBD/11015 for EA and CP, respectively in the herbarium of the institute. Aerial parts of EA and CP were dried in shade at room temperature. The shade dried plant material was coarsely powdered and subjected to extraction with petroleum ether in a Soxhlet apparatus. The extraction was continued till the defatting of the material had taken place. The defatted marc was then extracted with ethanol (95%) till complete extraction.

A preliminary analysis was carried out to determine the wavelength at which maximum intensity was shown by pure scopoletin. For this purpose, 100 μg/ml solution of pure scopoletin was prepared in distilled water. A range of excitation and emission wavelength was measured with scopoletin solution to get λ_max_. The λ_max_ shown by scopoletin was 435 nm. So a range of 430-460 nm was selected so as minimize the effects of pH, solvent polarity and molecular rigidity on the fluorescence maxima.

The aqueous extracts of EA and CP were scanned in the spectrofluorimeter in concentrations of 0.1 μg/ml and 0.01 μg/ml. The scanning was performed at excitation and emission wavelengths of 300 nm to 700 nm and absorption maxima were found at 435 nm. This wavelength corresponds to λ_max_ of scopoletin. No other absorption peaks were detected in the range screened except at 605 nm. Scanning between 430 to 460 nm did not exhibit any other peak. The results of this analysis are shown in Table 1

**TABLE 1:** SPECTROFLUORIMETRIC SCANNING OF THE AQUEOUS EXTRACTS OF *E. ALSINOIDES* AND *C. PLURICAULIS*

Drug sample	Concentration of extract (μg/ml)	Wavelength (nm)	Intensity
*E. alsinoides*	0.1	435	221.838
		605	36.939
*E. alsinoides*	0.01	435	120.680
		605	26.696
*C. pluricaulis*	0.1	435	455.239
		605	59.372
*C. pluricaulis*	0.01	435	88.645
		605	33.306

λ_max_ excitation - 300 nm and λ_max_ emission - 700 nm. Intensity was calculated by extrapolation from the standard curve

Standard curve of scopoletin was prepared in distilled water. First of all, stock solution containing 1000 μg per ml of scopoletin was prepared in distilled water. Then this stock solution was used for preparing required dilutions containing 5-30 μg/ml of scopoletin. The quantity of stock solution used was 0.5, 1.0, 1.5, 2.0, 2.5 and 3.0 ml, respectively. These samples were analyzed in the spectrofluorimeter against solvent blank (distilled water). The wavelength and intensity for each sample was recorded and standard curve was prepared between concentration and intensity of fluorescence. The equation of line for the standard curve was y = 34.642× and correlation coefficient (r) value was 0.9956.

Ten milligrams of ethanol extract was weighed accurately and dissolved in 10 ml of distilled water with vigorous shaking. It was filtered and volume made to 100 ml in distilled water. These solutions were analyzed in the spectrofluorimeter at 435 nm and intensity of fluorescence was recorded. Further, concentration of scopoletin in the extract samples was determined from the standard curve.

For the estimation of total coumarins calculated as scopoletin in crude drug samples, 1 g shade dried powdered crude drug sample of EA and CP was taken. It was subjected to extraction with cold water for a period of 30 min. The aqueous extract was obtained after filtration. The aqueous extract was taken in a volumetric flask and volume was made up to 100 ml with distilled water. The intensity of this diluted sample was determined using a spectrofluorimeter. The whole procedure was repeated thrice to get triplicate readings.

The whole procedure was again performed with 1 g samples of crude drugs i.e. EA and CP. Extraction was done with boiling water for a period of 30 min. The hot extracts were made up to volume in 100 ml volumetric flasks. The intensity of these diluted samples was determined spectrofluorimetrically. The procedure was also repeated thrice to get readings in triplicate.

After the spectrofluorimetric analysis by cold and hot extraction methods, the concentration of total coumarins calculated as scopoletin in all the samples was found out by extrapolating from the standard curve. Mean concentration of total coumarins calculated as scopoletin, present in 1 g of crude drug, was thus determined.

After determining the concentration of total coumarins calculated as scopoletin per ml of the aqueous extract, the mean concentration per gram of crude drug (% w/w) was calculated. The % yield per gram of crude drug powder of EA and CP was found to be as shown in the Table 2.

**TABLE 2:** PERCENT YIELDS OF TOTAL COUMARINS CALCULATED AS SCOPOLETIN (% W/W) IN *E. ALSINOIDES* AND *C. PLURICAULIS*

Drug sample	Method of extraction	Mean concentration (mg/ml)	% Yield (g/100 g of crude drug)
*E. alsinoides*	Cold extraction	0.018±0.008	0.0018
*E. alsinoides*	Hot extraction	0.020±0.021	0.0020
*C. pluricaulis*	Cold extraction	0.017±0.010	0.0017
*C. pluricaulis*	Hot extraction	0.023±0.004	0.0023

All values are mean±SEM (n=3)

It was observed that in the hot extraction method, the yield of total coumarins calculated as scopoletin increased to 12.80% w/w and 26.51% w/w for EA and CP, respectively. Thus a simple spectrofluorimetric method for analysis of total coumarins calculated as scopoletin in *shankhpushpi* was developed. Further recovery studies were performed for the validation of this novel analytical method.

Standard solutions (5-30 μg/ml) were prepared in distilled water and intensity of fluorescence was recorded in the spectrofluorimeter. The standard curve was prepared by plotting concentration as abscissa versus intensity of fluorescence as ordinate. Linear dependence of intensity on concentration was observed throughout the concentration range tested.

The precision of the method was checked for standard solutions of the aqueous extract at a concentration of 0.1 μg/ml, 0.2 μg/ml, 0.5 μg/ml and 1.0 μg/ml, prepared by appropriate dilutions with distilled water. The solutions were analyzed in a spectrofluorimeter at 430-460 nm and the intensity was recorded. The corresponding concentration was extrapolated from the standard curve.

The whole procedure was repeated thrice for each dilution and the readings were expressed as Mean±SEM (n=3). Then a 0.1 μg/ml solution of scopoletin was prepared by appropriate dilutions and analyzed spectrofluorimetrically. The concentration of scopoletin was calculated for the sample.

This sample of known concentration was added in equal volume (1 ml) to all the previous dilutions, and analyzed to see whether the practical concentration obtained is in correspondence with the theoretical or hypothetical concentration from the standard curve. Percentage recoveries were calculated on the basis of determination of analyte added to a sample containing a known amount of scopoletin (Table 3).

**TABLE 3:** VALIDATION OF THE SPECTROFLUORIMETRIC METHOD AND CALCULATION OF THE PERCENTAGE RECOVERY OF SCOPOLETIN

Concentration of aqueous extract used (μg/ml)	Scopoletin* content in extract (mg/ml)	Scopoletin added (mg/ml)	Total amount of scopoletin expected (mg/ml)	Amount obtained (mg/ml)	Percentage recovery (%)
0.1	0.009±.0.002	0.009	0.018±0.002	0.018±0.030	100.69±0.087
0.2	0.013±0.002	0.009	0.022±0.002	0.022±0.007	100.12±0.068
0.5	0.018±0.000	0.009	0.027±0.000	0.029±0.022	101.60±0.056
1.0	0.020±0.001	0.009	0.029±0.001	0.030±0.001	100.58±0.023

* As calculated from the standard curve. All values are mean±SEM (n = 3). ANOVA followed by student's t-test. *p*<0.001 was considered to be statistically significant.

Once the standard curve for scopoletin was prepared using a series of standard dilutions from 5 μg/ml to 30 μg/ml, the dilutions covering the detection limit of the instrument, it became feasible to estimate scopoletin in the herbal extracts by measuring their fluorescence intensity within the range of excitation and emission wavelengths for scopoletin, i.e. 430 to 460 nm. After the success of spectrofluorimetric analysis in determining the concentration of scopoletin in various extracts of EA and CP, it was thought worthwhile to develop a method for the determination of scopoletin concentration in crude drug samples.

Thus a simple analytical method was developed which proved to be very crucial in estimating concentration of total coumarins calculated as scopoletin in various drug samples. The developed method was validated for specificity, reproducibility and accuracy. The method was found to be specific for total coumarins calculated as scopoletin since the fluorescence maxima at λ_max_ 435 nm was identical for both the standard scopoletin and the scopoletin present in the extracts. Linearity range was found to be in the range of 5-30 μg/ml. The correlation coefficient (r) was 0.9956 indicating good linearity between fluorescence intensity and concentration. Repeated scanning of the samples three times checked precision of the method. Carrying out a recovery study confirmed reproducibility and accuracy of the method. A known concentration of scopoletin was added to varying concentrations of the aqueous extract i.e. 0.1, 0.2, 0.5 and 1.0 μg/ml. The sample of known concentration was added in equal volume to the various dilutions of the extract and analyzed spectrofluorimetrically to see whether the practical concentration obtained is in correspondence with the theoretical or hypothetical concentration from the standard curve. The percentage recovery for scopoletin was found to be in the range of 100-101%. Hence this developed spectrofluorimetric method is quick and reliable for quantitative monitoring of total coumarins calculated as scopoletin in raw material, processed powder and in herbal preparations containing EA and CP.

## References

[1] Nahata A, Patil UK, Dixit VK (2008). Effect of *Convulvulus pluricaulis* Choisy. on learning behaviour and memory enhancement activity in rodents. Nat Prod Res.

[2] Gupta AK, Tandon N, Sharma M (2005). Quality standards of Indian Medicinal Plants.

[3] Evans WC (1991). Farmacognosia.

[4] Murray RDH, Mendez J, Brown SA (1982). The natural coumarins. Occurance, Chemistry and Biochemistry.

[5] Fernandez IME, Quesada RJ, Villalon MM, Lopez MMC (2000). Comparison of methods for determining coumarins in distilled beverages. Food Chem.

[6] Harborne JB (1973). Phytochemical Methods: A guide to modern techniques of plant analysis.

[7] Otsuka K, Zenibayashi Y (1974). On the determination of scopoletin in aged distilled liquors. Agri Biol Chem.

[8] Ira N (1993). Fisicoquimica.

